# NIR-II Upconversion Photoluminescence of Er^3+^ Doped LiYF_4_ and NaY(Gd)F_4_ Core-Shell Nanoparticles

**DOI:** 10.3389/fchem.2021.690833

**Published:** 2021-05-31

**Authors:** Qilong Feng, Wenjing Zheng, Jie Pu, Qiaoli Chen, Wei Shao

**Affiliations:** College of Chemical Engineering and State Key Laboratory Breeding Base of Green Chemistry Synthesis Technology, Zhejiang University of Technology, Hangzhou, China

**Keywords:** upconversion, NIR-II, core-shell, Er^3+^, luminescence

## Abstract

The availability of colloidal nano-materials with high efficiency, stability, and non-toxicity in the near infrared-II range is beneficial for biological diagnosis and therapy. Rare earth doped nanoparticles are ideal luminescent agents for bio-applications in the near infrared-II range due to the abundant energy level distribution. Among them, both excitation and emission range of Er^3+^ ions can be tuned into second biological window range. Herein, we report the synthesis of ∼15 nm LiYF_4_, NaYF_4_, and NaGdF_4_ nanoparticles doped with Er^3+^ ions and their core-shell structures. The luminescent properties are compared, showing that Er^3+^ ions with single-doped LiYF_4_ and NaYF_4_ nanoparticles generate stronger luminescence than Er^3+^ ions with doped NaGdF_4_, despite the difference in relative intensity at different regions. By epitaxial growth an inert homogeneous protective layer, the surface luminescence of the core-shell structure is further enhanced by about 5.1 times, 6.5 times, and 167.7 times for LiYF_4_, NaYF_4_, and NaGdF_4_, respectively. The excellent luminescence in both visible and NIR range of these core-shell nanoparticles makes them potential candidate for bio-applications.

## Introduction

The 4f-4f transition of rare earth elements endows them with outstanding optical properties. Specifically, the energy of photons can be converted through rational designed transition among their abundant energy levels (Auzel, 2004; Wen et al., 2018). Furthermore, the photo-luminescence of rare earth is extremely stable, and it could not be affected by blinking or photobleaching (Shao et al., 2018; Song et al., 2019). These advantages make rare earth elements perfect candidates for light-triggered diagnostics and therapy (Chen et al., 2014; Shao et al., 2016; Qiang and Wang, 2019; Wang et al., 2020). As an unusual anti-stokes process, photon upconversion (UC) refers to converting several low-energy photons to one high-energy photon (Zhou et al., 2015; Wei et al., 2016). Due to the non-linear instincts, UC materials are well-developed for bio-imaging (Gonzalez-Bejar et al., 2016; Chen et al., 2021), photo-dynamics therapy(Lucky et al., 2015; Liu et al., 2019; Jia et al., 2020), and single particle imaging (Gargas et al., 2014; Liu et al., 2017; Zhou et al., 2020b; Dong et al., 2021).

The penetration depth of photons with different energies is significantly varied due to the scattering and absorption of biological tissue (Hemmer et al., 2016; Li and Wang, 2018). Compared to the visible range, where the auto-fluorescence of most tissue components is located, the near-infrared (NIR) range generally exhibits better spatial resolution and sharper contrast (Kenry et al., 2018). Recent studies have demonstrated that probes utilized in biological window II (1000–1700 nm, NIR-II) work more effectively than that in biological window I (700–900 nm, NIR-I) (He et al., 2015; Zhong et al., 2017; Fan et al., 2018; Huang et al., 2020; Lin et al., 2021; Lv et al., 2021; Yu et al., 2021). However, most reports on NIR probes are realized in the first biological window (Zhou et al., 2020a). Meanwhile, upconversion and down-shifting of rare earth with emission in biological window II have gained considerable interest recently. Zhong et al. have designed an Er-based nanoparticle (NPs) doped with Ce^3+^, which enhanced the down-shifting emission at 1550 nm under excitation at 980 nm (Zhong et al., 2017). They acquired high spatiotemporal resolution cerebral vasculatures in the NIR-II window with short exposure time. Liu et al. synthesized Er and Ho co-doped NPs with both excitation and emission in the NIR-II window as a sensor for inflammation dynamic detection (Liu et al., 2018). The time-gate imaging technique in the NIR-II window was realized in a NaYF_4_: Yb, Nd@CaF_2_ core shell structure with outstanding brightness (Tan et al., 2020). Er^3+^ ion is known as an outstanding imaging agent for NIR-II range, since it can be excited around 1550 nm due to the ^4^I_15/2_ → ^4^I_13/2_ transition (Chen et al., 2011; Shao et al., 2014; Cheng et al., 2018). Moreover, the emission peaks are at 550 nm (^4^S_3/2_ → ^4^I_15/2_) and 660 nm (^4^F_9/2_ → ^4^I_15/2_) in the visible range, 800 nm (^4^I_9/2_ → ^4^I_15/2_) in the NIR-I range, and 1000 nm (^4^I_11/2_ → ^4^I_15/2_) in the NIR-II range, respectively. The two-photon NIR-II to NIR-II UC process is rather unique, which have been investigated widely. However, how the matrix determines the luminescence remains unknown and deserves our efforts.

Herein, we reported the successful synthesis of three types of fluoride matrix, LiYF_4_, NaYF_4_, and NaGdF_4,_ each one doped with Er^3+^ ions. Their luminescence properties were compared, showing different relative intensity within each matrix. With epitaxial growth an inert homogeneous protect layer, the luminescence of each kind of UCNPs achieve much improvement due to the surface passivation. A proper mechanism was proposed based on the emission intensity dependence on excitation power density.

## Materials and Methods

### Materials

Yttrium (III) chloride hexahydrate (99.99%), erbium (III) chloride hexahydrate (99.9%), gadolinium (III) chloride hexahydrate (99.9%), yttrium (III) oxide (99.999%), gadolinium (III) oxide (99.9%), sodium trifluoroacetate (98%), lithium trifluoroacetate (95%), trifluoroacetic acid (98%), 1-octadecene (90%), and oleic acid (90%) were purchased from Sigma-Aldrich. Sodium hydroxide (98%), lithium hydroxide (98%), and ammonium fluoride (98%) were purchased from Aladdin Co. All chemicals were used as received.

### Instruments

The size and morphology of the resulting nanoparticles were characterized by Hitachi 7700 electron microscope. Upconversion emission spectra in visible and NIR range were recorded using Horiba Fluoromax–4. Laser excitation at 1532 nm, which was purchased from Changchun New Industries Optoelectronics Technology Co., Ltd. The emission signal from the sample in the cuvette was collected at 90° relative to the exciting laser beam. The powder X-ray diffraction (XRD) patterns were recorded by a Bruker D8 Advance diffractometer using Cu Kα radiation. The 2θ angle of the XRD spectra was recorded at a scanning rate of 5°/min.

### Synthesis of LiYF_4_:10%Er^3+^ (NaYF_4_:10%Er^3+^ and NaGdF_4_:10%Er^3+^)

In a typical procedure, a total of 0.1°mmol ErCl_3_•H_2_O and 0.9°mmol YCl_3_•H_2_O were added into a 100°ml flask containing 6°ml oleic acid and 15°ml octadecene. The mixture was heated to 160°C for 60°min then cooled down to room temperature. Subsequently, a solution of 4°mmol NH_4_F and 2.5°mmol LiOH (2.5°mmol NaOH for NaYF_4_:10%Er^3+^ and NaGdF_4_:10%Er^3+^) in 10°ml methanol was added and stirred for 30°min. The reaction mixture was then heated at 100°C for 30°min to remove the methanol, followed by heating up to 290°C (300°C for NaYF_4_:10%Er^3+^ and NaGdF_4_:10%Er^3+^) and keeping for 60°min before cooling down. A syringe needle was used to let the argon gas out during the synthesis. The mixture was cooled to room temperature and precipitated by excess ethanol and collected by centrifugation. The precipitate was washed with ethanol several times, and the nanocrystals were finally dispersed in hexane.

### Synthesis of LiYF_4_:10%Er^3+^@LiYF_4_ (NaYF_4_:10%Er^3+^@NaYF_4_, NaGdF_4_:10%Er^3+^@NaGdF_4_)

The LiYF_4_:10%Er^3+^@LiYF_4_ core-shell nanoparticles are synthesized via a thermal decomposition method. A total of 0.5°mmol of Y_2_O_3_ was dissolved in 50% trifluoroacetic acid at 95°C in a three-neck flask. Then, the solutions were evaporated to dryness under an argon gas purge. Next, 10°ml of oleic acid, 10°ml of 1-octadecene, 1.5°mmol lithium trifluoroacetate, and 1°mmol LiYF_4_:10%Er^3+^ core (2°mmol sodium trifluoroacetate and 1°mmol NaYF_4_:10%Er^3+^ core for NaYF_4_:10%Er^3+^@NaYF_4_, 2°mmol sodium trifluoroacetate and 1°mmol NaGdF_4_:10%Er^3+^ core for NaGdF_4_:10%Er^3+^@NaGdF_4_) were added into the flask. The resulting solution was then heated at 120°C with magnetic stirring for 45°min to remove water and oxygen. The brown solution was then heated to 310°C at a rate of about 12°C per min under argon gas protection and kept at this temperature under vigorous stirring for 45°min. A syringe needle was used to let the argon gas out during the synthesis. The mixture was cooled to room temperature and precipitated by excess ethanol and collected by centrifugation. The precipitate was washed with ethanol several times, and the nanocrystals were dispersed in hexane.

## Results and Discussion

**FIGURE 1:**
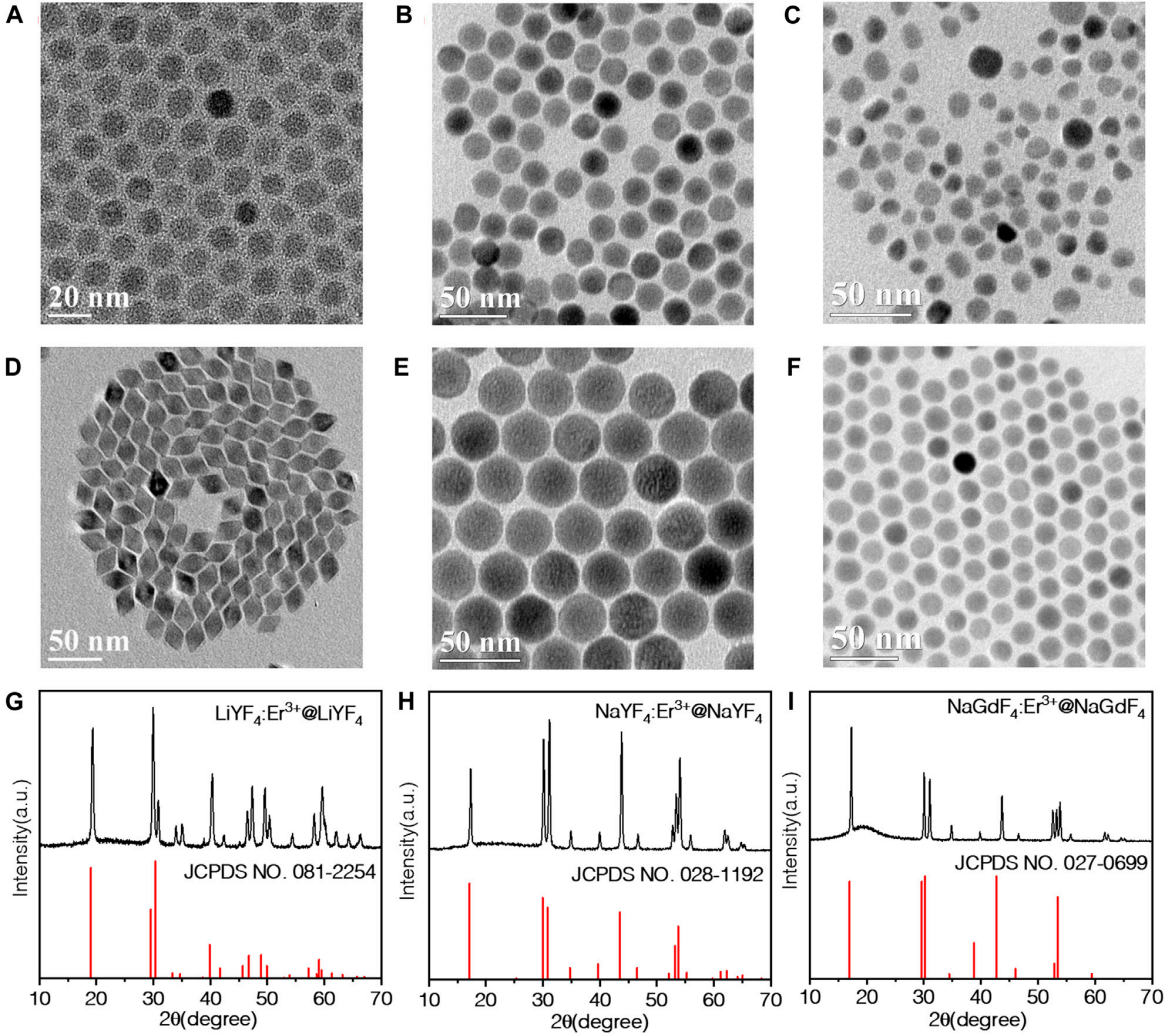
TEM images of **(A)** LiYF_4_:10%Er^3+^, **(B)** NaYF_4_:10%Er^3+^, **(C)** NaGdF_4_:10%Er^3+^, **(D)** LiYF_4_:10%Er^3+^@LiYF_4_, **(E)** NaYF_4_:10%Er^3+^@NaYF_4_, **(F)** NaGdF_4_:10%Er^3+^@NaGdF_4_ nanoparticles; and XRD pattern of **(G)** LiYF_4_:10%Er^3+^@LiYF_4_, **(H)** NaYF_4_:10%Er^3+^@NaYF_4_, **(I)** NaGdF_4_:10%Er^3+^@NaGdF_4_ nanoparticles.

In order to evaluate the emission intensity impacted by matrix, 10% Er^3+^ was doped into three matrix LiYF_4_, NaYF_4_ and NaGdF_4_ according to a previous synthesis procedure (Wang et al., 2010b). These three matrix types have been investigated extensively due to their low phonon energy (∼400°cm^–1^), which are beneficial for efficient UC luminescence. As shown in Figure 1, three types of UCNPs were all monodisperse in hexanes solvents. LiYF_4_ was shaped with spindle morphology (Figure 1A), while NaYF_4_ (Figure 1B) and NaGdF_4_ (Figure 1C) were shaped with the spheric shape, respectively. The size of LiYF_4_ is determined to be around 16 nm, NaYF_4_ around 19 nm, and NaGdF_4_ 14 nm (Supplementary Figures S1A–C), which makes them comparable since the luminescences of NPs are significantly effected by their sizes. The X-ray diffraction (XRD) pattern in Supplementary Figure S2 showed the LiYF_4_ possessed tetragonal phase, which was accordance with previous report. However, a hexagonal phase was identified for NaYF_4_, and a mixed hexagonal and cubic phase for NaGdF_4_ as the peaks at ∼27.5° and ∼32.5° referred to the (111) and (200) plane of the cubic phase of NaGdF_4_. Previous reports have concluded doping ions with larger size will efficiently reduce the energy and time for transformation from cubic to hexagonal phase (Wang et al., 2010a). Considering the reverse, a smaller size dopant is supposed to increase the energy barrier of transformation and reduce the stability of the hexagonal phase. In our case, the dopant size of Er^3+^ ion is 1.144 Å, which is smaller than Gd^3+^ (1.193 Å) and Y^3+^ (1.159 Å). The differential between Gd^3+^ and Er^3+^ is more remarkable than that of Y^3+^ and Er^3+^. As a result, the NaGdF_4_ requires more energy to complete the phase transfer. Then we further grew a homogeneous shell on each type of core UCNP as protect layer since the inert epitaxial shell growth could enhance the UC luminescence by overcoming the concentration quenching effects. The Transmission electron microscopy (TEM) imaging shows the sizes of three types of NPs grow larger, and the morphologies are much more uniform and monodisperse after coating the shell layer (Figure 1D–F). The size increase in the core-shell NPs is shown in Supplementary Figures S1D–F, and this proved the successful coating of shell on the core NPS. The phase of these core-shell UCNPs remain the same with core NPs, except NaGdF_4_ change from the fixed cubic and hexagonal phase to pure hexagonal phase (Supplementary Figures S1G–I). We believe the shell coating process provides enough energy for the phase transfer of NaGdF_4_ as well as epitaxial growth.

**FIGURE 2:**
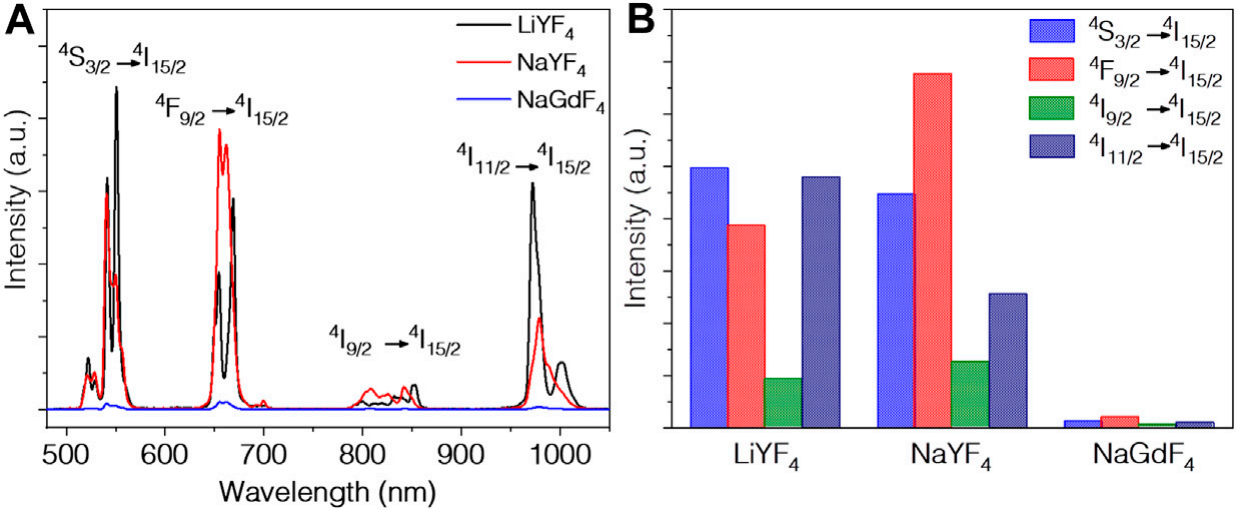
**(A)** UC emission spectra and **(B)** integration of all peaks of LiYF_4_:10%Er^3+^, NaYF_4_:10%Er^3+^, NaGdF_4_:10%Er^3+^ nanoparticles excited at 1532 nm.

The UC properties of three core NPs under 1532 nm excitation were compared. As shown in Figure 2, three types of core NPs all emit four characteristic peaks assigned to the ^4^S_3/2_ → ^4^I_15/2_ (550 nm), ^4^F_9/2_ → ^4^I_15/2_ (660 nm), ^4^I_9/2_ → ^4^I_15/2_ (800 nm), and ^4^I_11/2_ → ^4^I_15/2_ (1000 nm), respectively. The optimized concentration of Er^3+^ ions is determined to be 10% according to integration of the total emission intensity (Supplementary Figure S3). The emission intensity of NaYF_4_ core UCNPs is almost the same with LiYF_4_, which is about 50 times stronger than NaGdF_4_ UCNPs (Supplementary Figure S4). We further integrated the emission intensities of each peak for all three core NPs, respectively, as illustrated in Figure 2B. Notably, the ^4^F_9/2_ → ^4^I_15/2_ transition (660 nm) of NaYF_4_ is about 1.7 times stronger than LiYF_4_, while on the contrary, the ^4^I_11/2_ → ^4^I_15/2_ transition (1000 nm) of LiYF_4_ is about twice of the NaYF_4_. As we all know, the luminescence of rare earth can be ascribed to the local field splitting, which is highly depended on the crystal symmetry. Then it is understandable the tetragonal and hexagonal phases with different symmetry structures will favor variable energy transition, corresponding to the diverse dominant luminescence character. This explains the intensities of different peaks are not identical for the NaYF_4_ and LiYF_4_ matrix, and so the NIR-II emission bands of LiYF_4_:Er^3+^ are more suitable than NaYF_4_:Er^3+^ NPs. As for the NaGdF_4_, it contains both cubic and hexagonal phase. For the parity forbidden essence, the phase with lower symmetry exhibits stronger emission intensity than the phase with higher symmetry (Liu, 2015). The luminescence of hexagonal phase NaGdF_4_ is always much stronger than the cubic phase, so the mixed phase NaGdF_4_ emits less efficient than pure hexagonal phase NaYF_4_ (Damasco et al., 2014).

**FIGURE 3:**
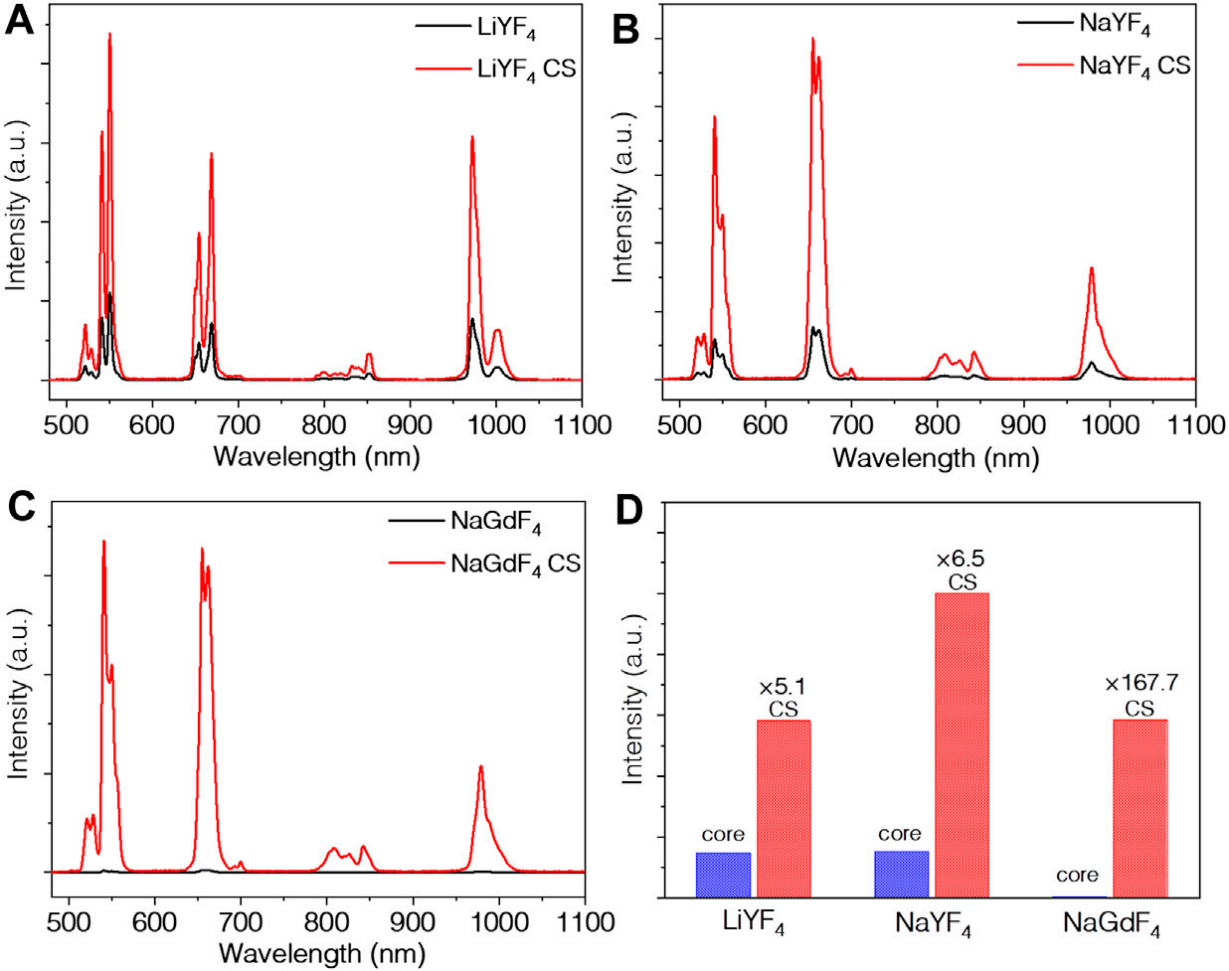
UC emission spectra of **(A)** LiYF_4_:10%Er^3+^ and LiYF_4_:10%Er^3+^@LiYF_4_, **(B)** NaYF_4_:10%Er^3+^ and NaYF_4_:10%Er^3+^@NaYF_4_, **(C)** NaGdF_4_:10%Er^3+^ and NaGdF_4_:10%Er^3+^@NaGdF_4_ nanoparticles, and **(D)** the integration of their emission peaks excited at 1532 nm.

For rare earth nanomaterials, the protection shell architecture can suppress the surface-related quenching effects. Especially for Er^3+^ heavily doped nanoparticles, the core-shell architecture can significantly enhance both the UC and down-shifting luminescence. The concentration quenching of bare core is contributed mainly by surface quenching of defects and high phonon ligand instead of common misconceptions of cross-relaxation of rare earth (Johnson et al., 2017). Indeed, by coating a homogeneous shell, the luminescent intensity is unanimously enhanced for all the UCNPs, specifically, 5.1 times for LiYF_4_, 6.5 times for NaYF_4_, and 167.7 times for NaGdF_4_, as shown in Figure 3. The enhancement of LiYF_4_ nanoparticles and NaYF_4_ nanoparticles is similar, which illustrates surface passivation is the dominant factor rather than the crystal phase, since the sizes of core LiYF_4_ and NaYF_4_ UCNPs are almost the same. Compared to LiYF_4_ and NaYF_4_, the increase in the luminescence of NaGdF_4_ is more remarkable. As mentioned above, the shell growth on NaGdF_4_ includes two processes: complete phase transfer from the mixed phase to pure hexagonal phase together with the epitaxial growth of protection shell. Two processes all result in considerable emission enhancement to the extent that the total luminescence of NaGdF_4_:Er^3+^@NaGdF_4_ is as high as that of LiYF_4_:Er^3+^@LiYF_4_ core-shell UCNPs. The luminescence of NaYF_4_:Er^3+^@NaYF_4_ is slightly higher than the other two types of UCNPs by about 1.6 times. Moreover, the emission intensity around 1000 nm of LiYF_4_:Er^3+^@LiYF_4_ core-shell UCNPs is slightly higher than NaYF_4_:Er^3+^@NaYF_4_ UCNPs. All three types of core-shell UCNPs exhibit excellent emission in visible and NIR range, showing great potential for NIR-II range bio-applications.

**FIGURE 4:**
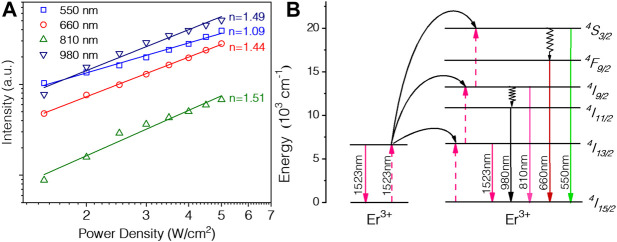
**(A)** Dependence of four peaks versus pump power, and **(B)** the proposed energy transfer upconversion mechanism between Er^3+^ ions.

Then, we measured the pump power dependences of four bands for the colloidal LiYF_4_:Er^3+^@LiYF_4_ core-shell UCNPs. As shown in Figure 4A, all the bands show a slope around 1, which is deviated from the reported UC mechanism. It is well established when the UC rate exceeds the decay rate for the intermediated states, the dependence on the excitation power P will decrease from P^n^ to P^1^. In our case, the saturation of ^4^I_13/2_ energy levels will cause such phenomenons, which explains the smaller measured photon numbers. These data are well accordance with previous literature, revealing similar energy transfer UC mechanism (Chen et al., 2011; Shao et al., 2014). Briefly, directly excitation of Er^3+^ ions by 1532 nm laser promotes the transition from ^4^I_15/2_ to ^4^I_13/2_. Then three-photon energy transfer process generates both green (^4^S_3/2_) and red (^4^F_9/2_) emission bands as well as the two-photon process of 800 nm (^4^I_9/2_) and 980 nm (^4^I_11/2_) emission peaks.

## Conclusion

In conclusion, we have observed four intensive emission bands for three colloidal and uniform LiYF_4_:Er^3+^@LiYF_4_, NaYF_4_:Er^3+^@NaYF_4_, and NaGdF_4_:Er^3+^@NaGdF_4_ core-shell UCNPs under excitation at 1532 nm. Homogeneous epitaxial growth of the shell layer promoted the luminescence intensity efficiently. Notably, NaGdF_4_ completed the phase transfer during the shell coating, which enhanced the core luminescence intensity about 167.7 folds. Taking LiYF_4_:Er^3+^@LiYF_4_ as example, the intensities of four UC peaks showed linear dependence on the excitation power, which can be explained by a saturation effect of ^4^I_13/2_ energy level. These core-shell UCNPs are pronounced as ideal NIR-II agent for biological related applications.

## Data Availability

The original contributions presented in the study are included in the article/Supplementary Material, further inquiries can be directed to the corresponding author.
