# Supplementary material for: NIR-II Upconversion Photoluminescence of Er3+ Doped LiYF4 and NaY(Gd)F4 Core-Shell Nanoparticles
Source: Front Chem. 2021 May 31;9:690833. doi: 10.3389/fchem.2021.690833 (PMC8201074; doi:10.3389/fchem.2021.690833)
Supplement: Supplementary file 1 [file DataSheet1.docx]

Supplementary Material

NIR-II Upconversion Photoluminescence of Er^3+^ Doped LiYF_4_ and NaY(Gd)F_4_ Core-Shell Nanoparticles

Qilong Feng^1†^, Wenjing Zheng^1†^, Jie Pu^1^, Qiaoli Chen^1^, Wei Shao^1*^

^1^ College of Chemical Engineering and State Key Laboratory Breeding Base of Green Chemistry Synthesis Technology, Zhejiang University of Technology, Hangzhou, China

*** Correspondence:**Corresponding Author: Wei Shao
weishao@zjut.edu.cn

† These authors contributed equally to this work

**Keywords: Upconversion, NIR-II, Core-Shell, Er^3+^, Luminescence.**


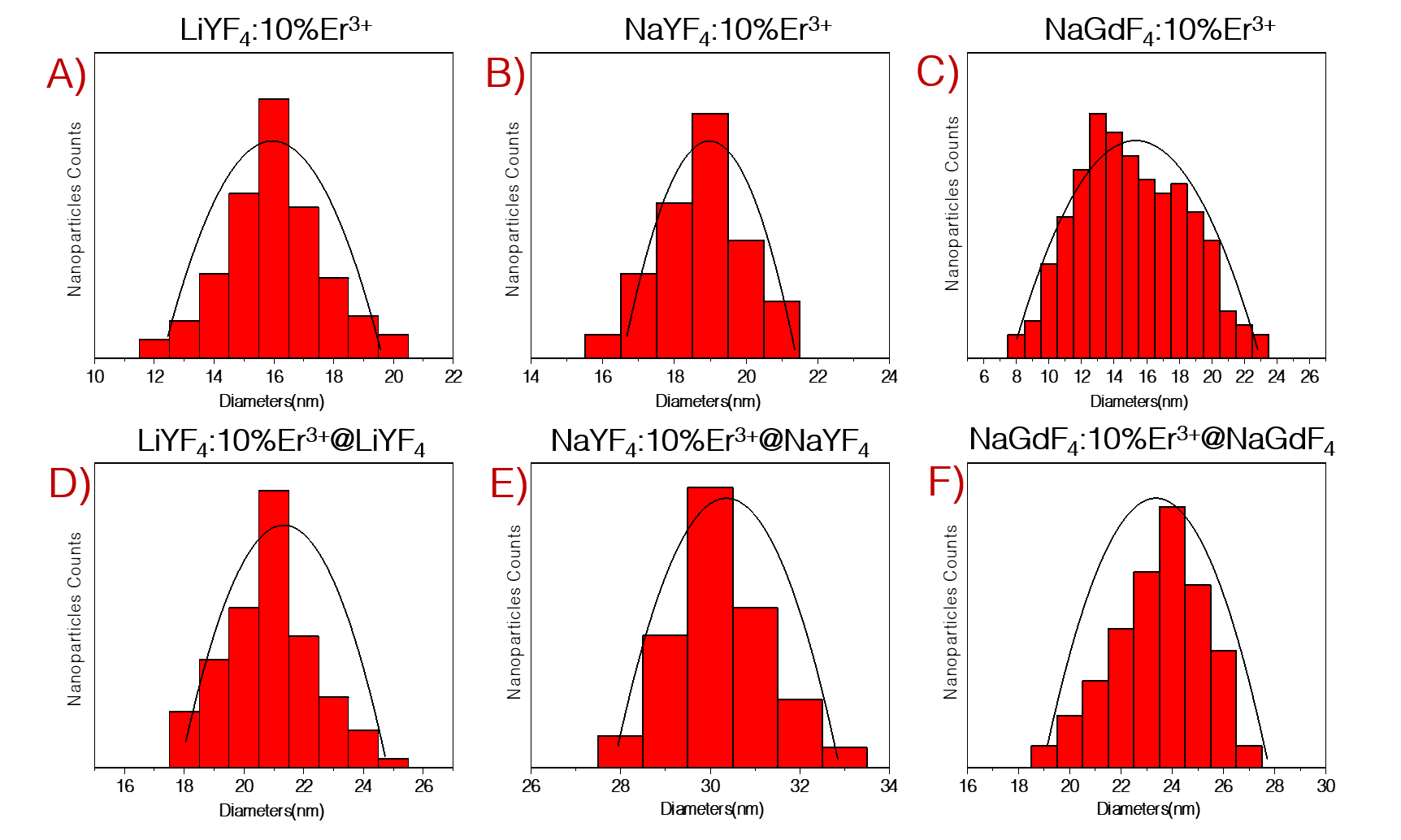


**Supplementary Figure S1**. Size distribution histograms of (A) LiYF_4_:10%Er^3+^, (B) NaYF_4_:10%Er^3+^, (C) NaGdF_4_:10%Er^3+^, (D) LiYF_4_:10%Er^3+^@LiYF_4_, (E) NaYF_4_:10%Er^3+^@NaYF_4_, (F) NaGdF_4_:10%Er^3+^@NaGdF_4_ nanoparticles.


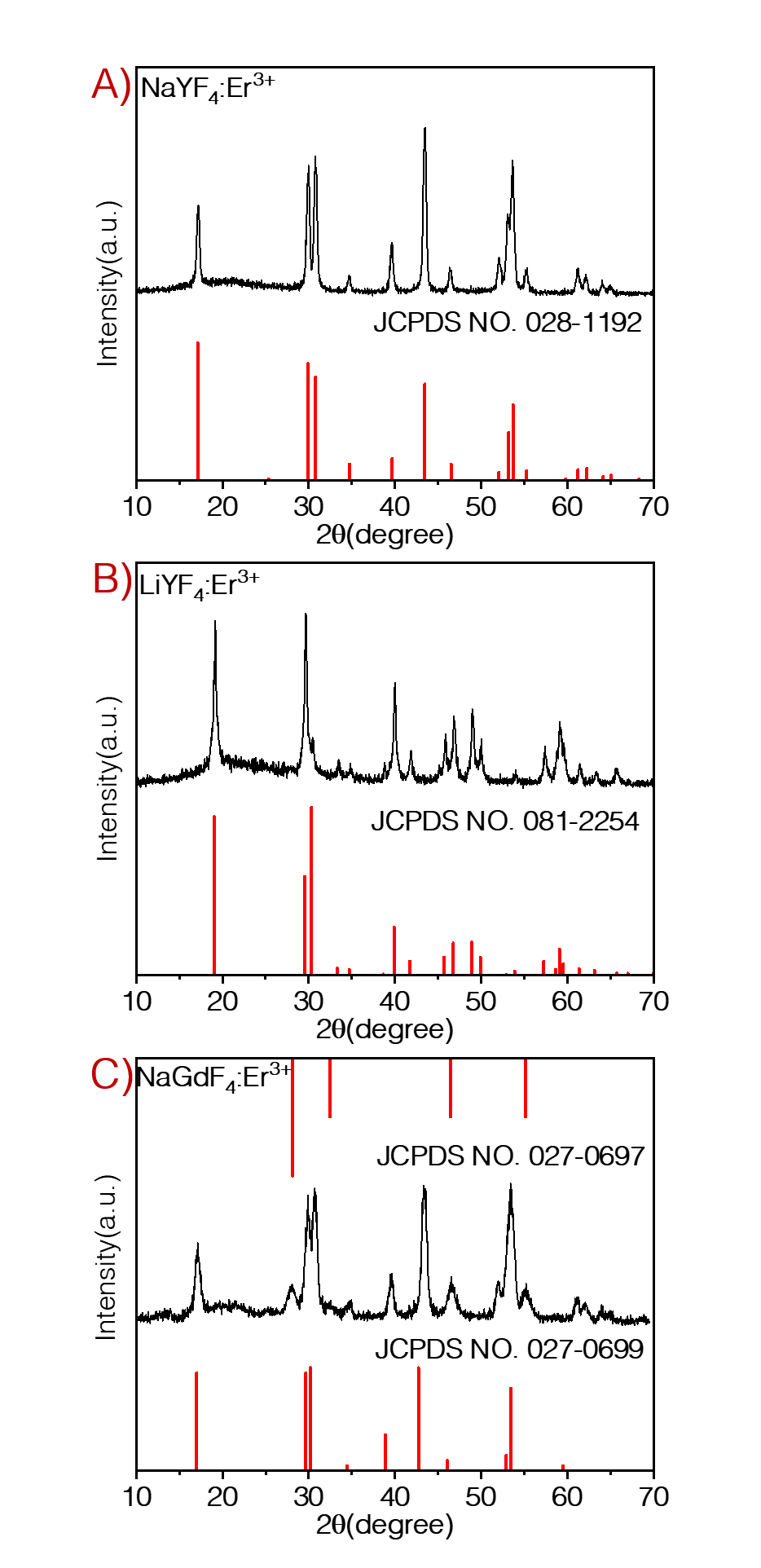


**Supplementary Figure S2**. The XRD pattern of (A) NaYF_4_:10%Er^3+^, (B)LiYF_4_:10%Er^3+^ and (C) NaGdF_4_:10%Er^3+^ nanoparticles.


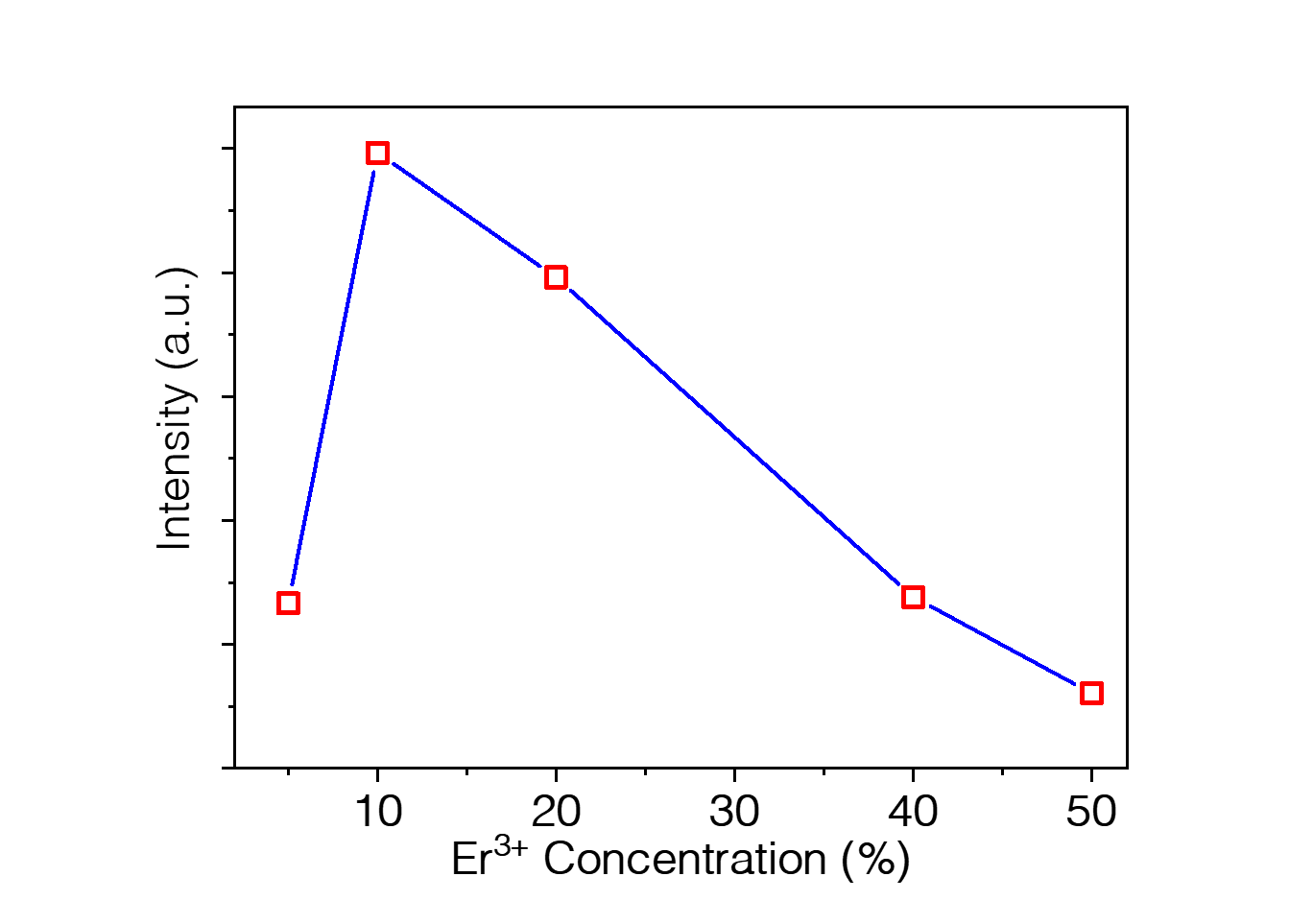


**Supplementary Figure S3**. The integrated upconversion emission intensity of LiYF_4_:Er^3+^ core nanoparticles doped with different Er^3+^ concentration.


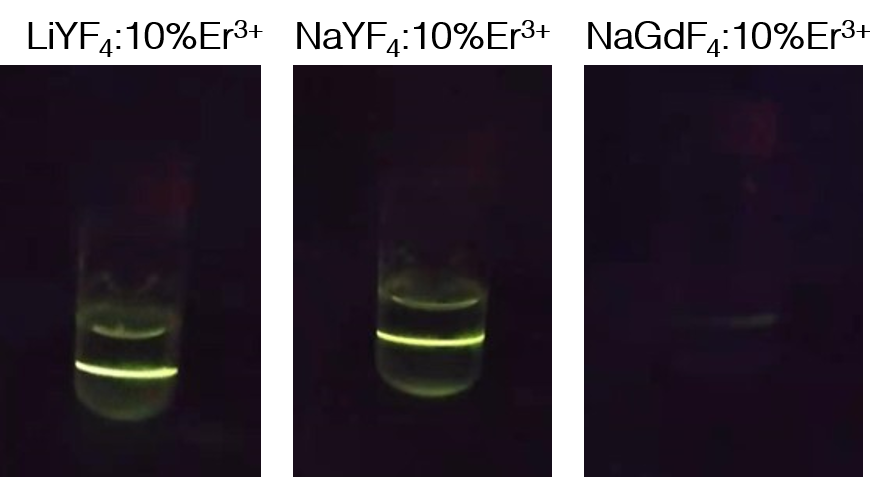


**Supplementary Figure S4**. Digital photos of NaYF_4_:10%Er^3+^, LiYF_4_:10%Er^3+^ and NaGdF_4_:10%Er^3+^ nanoparticles excited at 1532 nm laser.
